# The green tea catechin EGCG provides proof-of-concept for a pan-coronavirus attachment inhibitor

**DOI:** 10.1038/s41598-022-17088-0

**Published:** 2022-07-28

**Authors:** Emmanuelle V. LeBlanc, Che C. Colpitts

**Affiliations:** Department of Biomedical and Molecular Sciences, Queen’s University, Kingston, ON K7L 3N6 Canada

**Keywords:** SARS-CoV-2, Antivirals, Virus-host interactions

## Abstract

The COVID-19 pandemic caused by the severe acute respiratory syndrome coronavirus-2 (SARS-CoV-2) has emphasized the serious threat to human health posed by emerging coronaviruses. Effective broadly-acting antiviral countermeasures are urgently needed to prepare for future emerging CoVs, as vaccine development is not compatible with a rapid response to a newly emerging virus. The green tea catechin, epigallocatechin gallate (EGCG), has broad-spectrum antiviral activity, although its mechanisms against coronavirus (CoV) infection have remained unclear. Here, we show that EGCG prevents human and murine CoV infection and blocks the entry of lentiviral particles pseudotyped with spike proteins from bat or highly pathogenic CoVs, including SARS-CoV-2 variants of concern, in lung epithelial cells. Mechanistically, EGCG treatment reduces CoV attachment to target cell surfaces by interfering with attachment to cell-surface glycans. Heparan sulfate proteoglycans are a required attachment factor for SARS-CoV-2 and are shown here to be important in endemic HCoV-OC43 infection. We show that EGCG can compete with heparin, a heparan sulfate analog, for virion binding. Our results highlight heparan sulfate as a conserved cell attachment factor for CoVs, and demonstrate the potential for the development of pan-coronavirus attachment inhibitors, which may be useful to protect against future emerging CoVs.

## Introduction

The novel severe acute respiratory syndrome coronavirus-2 (SARS-CoV-2) has infected hundreds of millions of people and has resulted in millions of deaths around the world. The COVID-19 pandemic, caused by SARS-CoV-2, underscores the severe health threat posed by emerging coronaviruses (CoVs), and makes evident the paucity of antivirals with extended spectrums of activity. SARS-CoV-2 is the third highly pathogenic CoV to emerge in the twenty-first century, and the abundance and diversity of SARS-related CoVs in bats points to the likelihood of future spillover into human populations^1^. In addition, seasonally circulating common cold CoVs continually burden human health^2^, and cause severe disease in elderly populations^3,4^, without effective preventative or therapeutic strategies. Antivirals that could be administered prophylactically and that are effective against this broad range of endemic, pandemic, and emerging CoVs are urgently required. However, inhibitors with pan-coronavirus antiviral activity have not yet been developed.

Epigallocatechin gallate (EGCG) is the most bioactive polyphenolic compound from green tea with antiviral activity against diverse DNA and RNA viruses, including herpes simplex virus type 1 (HSV-1), hepatitis C virus (HCV), and influenza A virus (IAV)^5,6^. In the context of CoVs, molecular docking studies and in vitro binding and enzyme activity assays suggest that EGCG could inhibit the 3C-like protease of SARS-CoV-2 and other human CoVs^7–9^. EGCG was demonstrated to inhibit authentic SARS-CoV-2 virus infection in vitro when added early during infection ^10^, although the mechanisms remained unclear. The activity of EGCG against SARS-CoV-2 has been attributed, at least in part, to disrupting binding to its receptor, angiotensin-converting enzyme 2 (ACE2)^10,11^. Interestingly, EGCG was also shown to inhibit HCoV-OC43 infection^8,11,12^. The in vivo efficacy of EGCG was highlighted in a murine model of HCoV-OC43 infection in which EGCG-fed mice had reduced viral burden in the lungs compared to untreated mice^12^. However, HCoV-OC43 does not bind the ACE2 receptor (a proposed target of EGCG against SARS-CoV-2^10,11^), suggesting the involvement of alternative or additional antiviral mechanisms against CoVs, which are still unknown. Therefore, we sought to determine the spectrum of activity of EGCG against endemic, pandemic, and pre-emergent CoVs, and to understand the underlying broad antiviral mechanism(s).

Here, we have evaluated and characterized the inhibitory activity of EGCG against entry of human seasonal and highly pathogenic CoVs, as well as murine and bat CoVs such as WIV1-CoV, which is poised for future emergence into humans^13^. We demonstrate that EGCG inhibits entry of a broad range of CoVs into physiologically relevant human lung epithelial cells. Furthermore, EGCG inhibits binding of multiple human CoVs to cell surfaces, suggesting that this natural product inhibits a highly conserved step in CoV attachment, such as primary attachment to cell-surface heparan sulfate. We confirm the importance of cell surface heparan sulfate (HS) in promoting SARS-CoV-2 infection^14–16^ and reveal that that HS also contributes to HCoV-OC43 attachment. Focusing on SARS-CoV-2, we show that EGCG competetively inhibits virion attachment to heparin, a structural analog of HS. These findings further our understanding of the antiviral mechanisms of EGCG against CoVs, and identify a highly conserved antiviral target for the development of improved antiviral molecules to prevent infection by diverse CoVs, including potential future emerging CoVs.

## Results

### EGCG inhibits murine and human coronavirus infection

**Figure 1:**
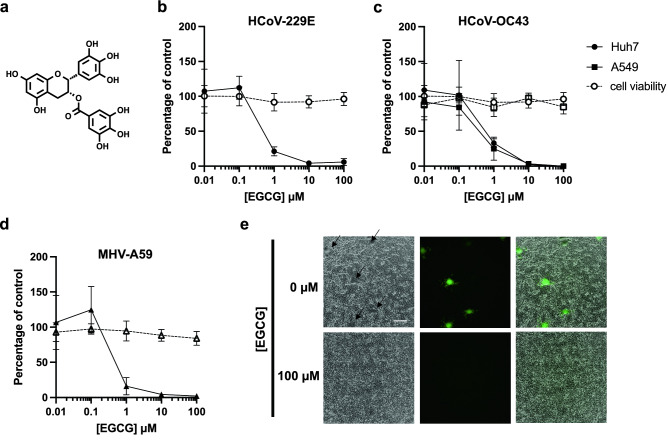
EGCG pre-treatment inhibits infection by diverse CoVs without affecting cell viability. (**a**) Structure of epigallocatechin gallate (EGCG). (**b-d**) Pre-treatment of authentic HCoV-229E, HCoV-OC43 or murine coronavirus MHV-A59-GFP virions with EGCG for 10 min at 37 °C inhibits infection of Huh7 **(b,c)**, A549 **(c)** or L929 cells **(d)**, respectively. Mean values with standard deviation from three independent experiments are plotted. Cell exposure to EGCG during the 2 h infection had minimal effect on cell viability (dashed line), where mean values with standard deviation of two independent experiments with duplicates are plotted. (**e**) A reduction in syncytia (black arrows) was observed for EGCG-treated MHV-A59. Scale bar: 200 μm.

We first tested the effect of EGCG (Fig. 1a) on the infectivity of two seasonal human CoVs, HCoV-229E (an alphacoronavirus) and HCoV-OC43 (a betacoronavirus). HCoV-229E and HCoV-OC43 virions were treated with DMSO or varying concentrations of EGCG for 10 min at 37 °C and used to inoculate infection-susceptible human hepatoma Huh7 or human lung epithelial A549 cell monolayers. After 2 h of infection, inocula were removed and cells were overlaid with plaquing media. Infectivity was assessed by plaquing efficiency on Huh7 cells for HCoV-229E or by immunofluorescence focus-forming assay on Huh7 and A549 cells for HCoV-OC43. EGCG inhibited infectivity of HCoV-229E (IC_50_ = 0.77 μM) and HCoV-OC43 (IC_50_ = 0.49–0.56 μM) at sub- to low micromolar concentrations (Fig. 1b,c), similar to other viruses susceptible to inhibition by EGCG, such as HSV-1^6^. We next tested the inhibitory activity of EGCG against a murine CoV, murine hepatitis virus (MHV-A59), on murine fibroblast L929 cell monolayers. We observed that EGCG inhibited MHV-A59 infection with similar potency as for the endemic human CoVs (IC_50_ = 0.71 μM) (Fig. 1b–d) and inhibited the formation of syncytia (Fig. 1e). Consistent with previous reports, we found EGCG to have minimal effect on cell viability at the low micromolar range in which antiviral activity is exerted (Fig. 1b–d). The finding that EGCG very similarly inhibits infectivity of these diverse CoVs, which utilize distinct receptors and entry mechanisms, suggests that it is targeting a highly conserved infection step.

### Entry of highly pathogenic coronaviruses is blocked by EGCG

**Figure 2:**
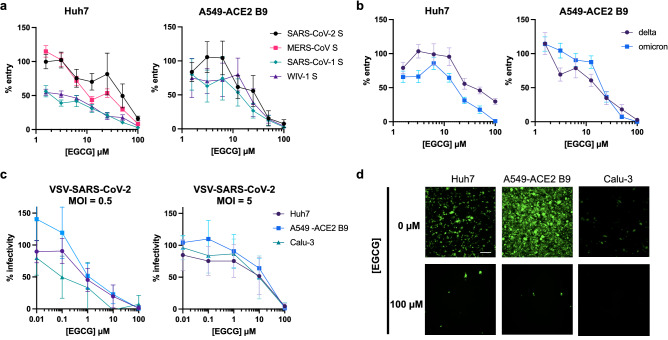
EGCG inhibits entry mediated by spike of highly pathogenic CoVs and a pre-emergent bat CoV. (**a-b**) EGCG pre-treatment reduced entry of SARS-CoV-1, MERS-CoV, SARS-CoV-2 (ancestral, delta or omicron), or WIV1-CoV pseudoparticles into Huh7 or A549-ACE2 cells. Mean values with standard error of the mean from three independent experiments each done in triplicate are plotted. (**c**) EGCG inhibits VSV-SARS-CoV-2 infection in Huh7, A549-ACE2 B9 and Calu-3 cells. Its antiviral potency is dependent on the multiplicity of infection (MOI). Mean values with standard deviation of three independent experiments each in duplicate are plotted. (**d**) Representative images are shown. Scale bar: 200 μm.

We next evaluated the activity of EGCG against entry of highly pathogenic betacoronaviruses. We produced lentiviral particles pseudotyped with SARS-CoV-1, SARS-CoV-2 and MERS-CoV spike (S) proteins to investigate the effect of EGCG on entry of highly pathogenic CoVs. We also generated lentiviral particles pseudotyped with the spike protein of WIV1-CoV, a bat coronavirus that binds to human ACE2^17^. CoV pseudoparticles were pre-exposed to EGCG or DMSO for 10 min at 37 °C, and then added to Huh7 cells. Inocula were removed after 2 h and replaced with fresh DMEM. After 72 h, cells were lysed and luminescence was measured. EGCG inhibited SARS-CoV-1, SARS-CoV-2, MERS-CoV and WIV1-CoV pseudoparticle entry with IC_50_ of 2.5, 48.6, 13.7 and 3.8 μM, respectively (Fig. 2a). To evaluate the antiviral effect of EGCG in a more relevant cell type, we generated A549 cells stably expressing human ACE2, the receptor for SARS-CoV-1 and SARS-CoV-2, and isolated a clone (B9) with high levels of ACE2 expression and enhanced susceptibility to SARS-CoV-2 S pseudoparticle infection, but not HCoV-OC43 infection which does not require ACE2 as a receptor (Fig. S1a–e). We found that EGCG also inhibited entry of SARS-CoV-1, SARS-CoV-2, and WIV1-CoV pseudoparticles into A549-ACE2 B9 cells, with IC_50_ of 15.9, 24.0 and 24.5 μM, respectively (Fig. 2a). Furthermore, we confirmed that the inhibitory activity of EGCG is maintained against lentiviral particles pseudotyped with the spike of SARS-CoV-2 variants of concern (VOCs), delta (B.1.617.2v1) and omicron (B.1.1.529) (Fig. 2b).

We next assessed the effect of EGCG on replication-competent vesicular stomatitis virus (VSV) virions expressing GFP and SARS-CoV-2 S instead of the VSV glycoprotein (VSV-SARS-CoV-2)^18^. Huh7, A549-ACE2 B9, and lung epithelial Calu-3 cells were infected with EGCG-treated VSV-SARS-CoV-2 virions (multiplicity of infection (MOI) of 5). Infected cells were imaged by fluorescence microscopy after 24 h. Similar to the pseudoparticle assays, EGCG inhibited VSV-SARS-CoV-2 infection (Fig. 2c,d) with IC_50_ ranging from 11.45 to 15.40 μM, depending on the cell type, with minimal cytotoxicity up to 100 μM (Fig. S2a). We next evaluated whether the effect of EGCG was dependent on MOI. Using the same assay, but with an MOI of 0.5, we found that EGCG inhibited VSV-SARS-CoV-2 infection with IC_50_ ranging from 0.14 to 1.13 μM. These findings demonstrate that the inhibitory activity of EGCG depends on the MOI (Fig. 2c), consistent with the virions as the proposed target of EGCG^6^. This also provides a potential explanation for the discrepancy in IC_50_ for authentic viruses investigated at low MOI (< 0.001) (Fig. 1b–d) compared to lentiviral pseudoparticles (Fig. 2a,b), for which p24 ELISA data for lentiviral particles pseudotyped with SARS-CoV-2 spike in our laboratory provide an estimate of approximately 1.7 × 10^6^ transducing units per mL, resulting in an apparent MOI of 3.4. Overall, our findings show that EGCG similarly inhibits infectivity of diverse CoVs, including human seasonal CoVs, recently emerged highly pathogenic CoVs, pre-emergent bat CoVs, and rodent CoVs.

### EGCG acts on virions to inhibit coronavirus binding to cell surfaces

**Figure 3:**
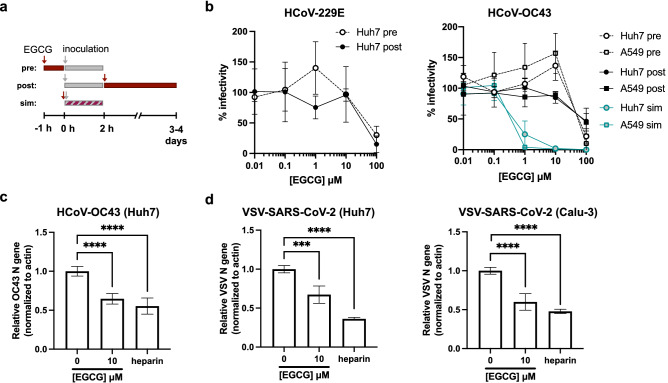
EGCG inhibits CoV attachment to cells. (**a**) Time-of-addition assay showing pre-treatment (pre), post-treatment (post) or simultaneous (sim) treatment of cells. **(b)** Pre-treatment of cells, or treatment of cells post-infection with HCoV-229E or HCoV-OC43, does not inhibit infection to similar extents as virion pre-treatment. Mean values with standard deviation of three independent experiments are plotted. (**c**) HCoV-OC43 virions were pre-treated with EGCG or heparin for 10 min at 37 °C then cooled on ice and added to pre-chilled Huh7 for 1 h on ice. (**d**) VSV-SARS-CoV-2 were pre-treated as described above and added to pre-chilled Huh7 or Calu-3 cells. Attached virions were quantified by RT-qPCR after washing cells with phosphate buffered saline (PBS) three times. Mean values with standard deviation from qPCR triplicates of two independent experiments are plotted. Statistical significance was assessed by Welch’s t test (***p < 0.001, ****p < 0.0001).

We next sought to understand the mechanism underlying the pan-CoV activity of EGCG. We first confirmed that EGCG acts on CoV virions, and not on a cellular target, by performing time-of-addition experiments (Fig. 3a). Cells were pre-treated with EGCG or DMSO 1 h at 37 °C, at which point media was removed and cells were infected with HCoV-229E or HCoV-OC43. Infectivity was determined by plaque assay or immunostaining. Alternatively, cells were infected, and EGCG- or DMSO-containing medium was added 2 h post-infection for the duration of the infection. Cell viability was minimally affected by prolonged EGCG treatment (Fig. S2b). In either condition, no inhibition of infectivity was observed until the highest concentration of 100 μM EGCG (Fig. 3b), in contrast to the inhibitory effect we observed when virions were pre-exposed to EGCG (Fig. 1b,c), or when EGCG was added at the same time as HCoV-OC43 (Fig. 3b), suggesting that EGCG acts at an early (likely extracellular) infection step. While we had previously established that EGCG does not affect the integrity or fluidity of virion envelopes^6^, we next sought to determine whether EGCG modifies cellular membranes by inducing phospholipidosis, which has confounded drug repurposing efforts for SARS-CoV-2^19^. We observed that EGCG did not induce phospholipidosis (Fig. S3), suggesting that it likely exerts specific inhibitory effects against a CoV entry step.

To test if EGCG inhibits CoV attachment to cell surfaces, we performed binding assays in which cells were incubated with pre-treated virions at 4 °C for 1 h to allow for attachment, but not fusion or entry. After cell washing, bound virions were quantified by RT-qPCR using primers specific for the viral N gene (Fig. S4). Pre-chilled Huh7 or Calu-3 cells were inoculated with HCoV-OC43 or VSV-SARS-CoV-2 virions that had been pre-treated with DMSO vehicle, EGCG (10 μM), or heparin (20 μg/mL), the latter as a control attachment inhibitor^14^. We observed that EGCG similarly reduced HCoV-OC43 and VSV-SARS-CoV-2 binding to Huh7 and Calu-3 cells (Fig. 3c,d), despite the different entry receptors used by these viruses. The requirement of ACE2 in SARS-CoV-2 infection is well documented, and HCoV-OC43, unlike other CoVs, does not have a known protein receptor but is thought to depend on sialic acid for entry. We observed that heparin partially blocked VSV-SARS-CoV-2 binding, consistent with the described role of heparan sulfate in SARS-CoV-2 entry. Unexpectedly, heparin also reduced HCoV-OC43 binding, providing support for recent findings suggesting involvement of glycosaminoglycans in HCoV-OC43 attachment^20^ and entry^21^. These findings are consistent with EGCG disrupting conserved CoV attachment to heparan sulfate.

### Heparan sulfate is a shared co-factor for SARS-CoV-2 and HCoV-OC43 infection

**Figure 4:**
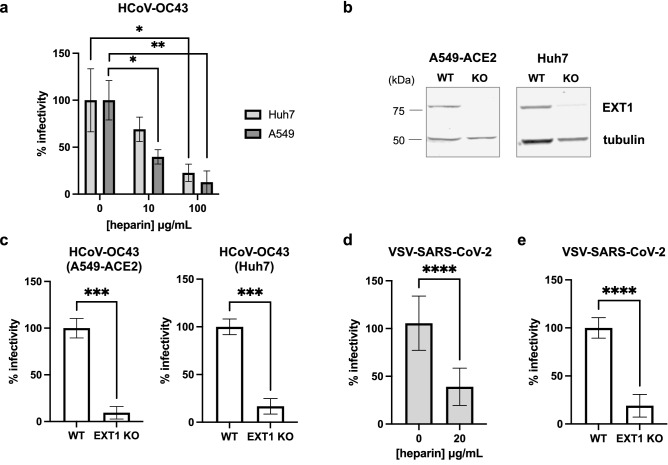
Heparan sulfate facilitates both SARS-CoV-2 and HCoV-OC43 infection. Pre-treatment of HCoV-OC43 (**a**) with heparin for 10 min at 37 °C reduced infection of Huh7 and A549 cells. (**b**) Western blot showing KO of EXT1 in A549-ACE2 and Huh7 cells. (**c**) HCoV-OC43 was titrated on WT and EXT1 KO cells, revealing reduced susceptibility of EXT1 KO cells to infection. (**d, e**) VSV-SARS-CoV-2 infection of A549-ACE2 cells is reduced by heparin pre-treatment or in the absence of EXT1. Mean values with standard deviation of three independent experiments are plotted. Statistical significance was assessed by Welch’s t test (*p < 0.05, **p < 0.01, ***p < 0.001, ****p < 0.0001).

To further probe the role of heparan sulfate in HCoV-OC43 infection, we evaluated if heparin not only reduced HCoV-OC43 cell surface binding (Fig. 3c) but also inhibited infection. First, HCoV-OC43 virions were treated with increasing concentrations of heparin for 10 min at 37 °C and used to inoculate Huh7 and A549 cells. Heparin dose dependently inhibited HCoV-OC43 infectivity (Fig. 4a). To rule out a non-specific inhibitory effect of heparin, we used CRISPR/Cas9 to knock out exostosin glycosyltransferase 1 (EXT1), a key enzyme involved in heparan sulfate biosynthesis, in Huh7 and A549-ACE2 cells (Fig. 4b). Interestingly, deletion of EXT1 substantially reduced the susceptibility of A549-ACE2 or Huh7 cells to HCoV-OC43 infection (Fig. 4c), highlighting the underappreciated role of heparan sulfate in mediating HCoV-OC43 infection. We confirmed that heparin pre-treatment or the absence of EXT1 reduces VSV-SARS-CoV-2 infectivity (Fig. 4d,e), consistent with other published work^14^. These findings suggest heparan sulfate is a shared co-factor required for entry of highly pathogenic and endemic CoVs, leading us to propose that EGCG may be disrupting CoV attachment to cell surface heparan sulfate.

### EGCG disrupts binding of SARS-CoV-2 to heparin

**Figure 5:**
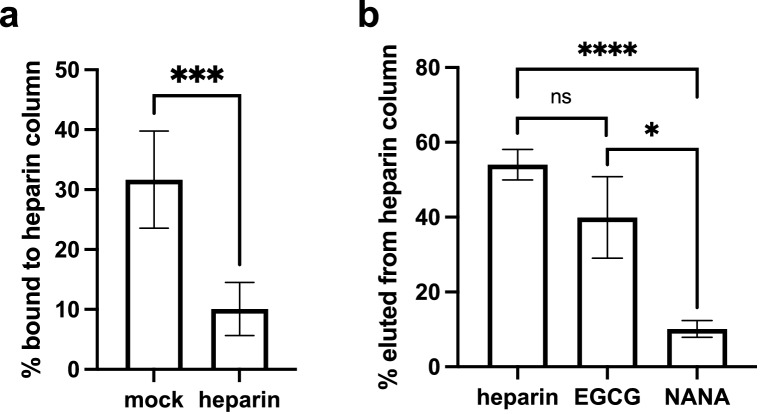
EGCG competes for SARS-CoV-2 heparin binding. (**a**) VSV-SARS-CoV-2 virions (1 × 10^5^ pfu) were pre-treated with 20 μg/mL heparin or mock and loaded onto a heparin column. After washing, still bound virions were then eluted with 2 M NaCl. Virions in flow through were quantified by viral RNA to determine percentage of bound virions before elution. **(b)** Alternatively, virions were loaded, and after washing, were eluted with 0.5 mg/mL heparin, EGCG, or NANA. Mean values with standard error of the mean of three independent experiments each with qPCR triplicates are plotted. Statistical significance was assessed by Welch’s t test (ns, not significant, *p < 0.05, ***p < 0.001, ****p < 0.0001).

Since EGCG reduced VSV-SARS-CoV-2 and HCoV-OC43 binding to cell surfaces to a similar extent as heparin, we tested the hypothesis that EGCG blocks the interaction of CoV spike proteins with heparan sulfate using heparin affinity chromatography. We first confirmed that VSV-SARS-CoV-2 virions bound to a heparin column, and that heparin could block binding (Fig. 5a). We then tested whether EGCG could competitively elute VSV-SARS-CoV-2 virions bound to heparin column. Virions were loaded and, after washing, were treated with 0.5 mg/mL EGCG, heparin as a positive control, or N-acetylneuraminic acid (NANA) as a negative control. Virions eluted by compound treatment were detected by quantifying viral RNA by RT-qPCR using primers specific for the VSV N gene. Heparin and EGCG, but not NANA, similarly competed virions off the column (Fig. 5b), consistent with the proposed mechanism where EGCG exerts its antiviral effect by disrupting CoV attachment to heparan sulfate. Furthermore, our data suggest that the combination of EGCG and heparin has an additive effect on inhibition of HCoV-OC43 infectivity, consistent with the two compounds acting by the same mechanism (Fig. S5).

## Discussion

Green tea catechins, especially EGCG, exert antiviral effects against diverse viruses^6^. We show that EGCG broadly inhibits infectivity of CoVs, and that its activity is due, at least in part, to blocking CoV primary attachment to target cells. Infection by authentic murine and human CoVs, as well as entry of lentivirus particles pseudotyped with spike proteins of highly pathogenic recently emerged or pre-emergent CoVs^17^, was inhibited by EGCG treatment. EGCG was recently shown to also inhibit authentic SARS-CoV-2 infection, and time-of-addition experiments suggested that EGCG at least partially blocks SARS-CoV-2 entry^10^, consistent with our findings. Furthermore, a recent study identified EGCG as an inhibitor of HCoV-OC43 infection in cell culture and murine models^12^, although the antiviral mechanisms were not explored in that study. Here, we expand the scope of EGCG activity to other CoVs, including murine and bat CoVs, and elucidate a mechanism explaining its pan-CoV antiviral activity.

A variety of mechanisms for the antiviral activity of EGCG have previously been described. EGCG inhibits the primary attachment of diverse viruses including VSV, IAV, HCV, HSV-1 and vaccinia virus^6^. EGCG is proposed to act directly on HCV virions to block attachment in a genotype independent manner, with no alteration in HCV (co)-receptor expression nor any effect on viral replication, assembly, or release^22,23^. As for hepatitis B virus (HBV), EGCG may inhibit entry^24^, as well as replication, with proposed roles in impairing HBV replicative intermediates during DNA synthesis^25^, diminishing the transcriptional activation of the HBV core promoter^26^, and enhancing lysosomal acidification to adversely affect HBV replication^27^. Alternatively, EGCG is suggested to prevent the binding of HIV glycoprotein 120 to its receptor molecule CD4 on cells^28^, and to act as an HIV reverse transcriptase inhibitor^29^. Finally, recent studies have identified in vitro inhibitory effects of EGCG against the SARS-CoV-2 3C-like protease^7–9,30^. However, our time-of-addition experiments with HCoV-229E and HCoV-OC43, as well as those of others with SARS-CoV-2^10^, strongly suggest that EGCG predominantly inhibits authentic CoV infection by blocking entry, although other activities might be exerted at higher concentrations. While some studies have proposed that EGCG inhibits interaction of the SARS-CoV-2 spike protein with ACE2^10,11^, this does not explain the ability of EGCG to inhibit the entry of diverse coronaviruses that utilize other receptors. We show that EGCG reduces cell surface binding of HCoV-OC43 and VSV-SARS-CoV-2 in a similar manner as heparin, which has been demonstrated to competitively block virion attachment^14^. We demonstrate that heparan sulfate facilitates VSV-SARS-CoV-2 and HCoV-OC43 infection, and that EGCG can competitively block virion binding to heparin.

Most human viruses, including CoVs, require low-affinity but high-avidity interactions with glycans to attach to cell surfaces and initiate infection. Heparan sulfate proteoglycans are necessary attachment receptors for SARS-CoV-2^14–16^ as well as the human alphacoronavirus NL63 (HCoV-NL63)^31^, as well as many other human viruses, such as HSV^32,33^, HCV^34,35^, and HIV^36^. Alternatively, other viruses, including influenza A virus (IAV) and betacoronaviruses HCoV-OC43 and HCoV-HKU1 are thought to use sialic acid-containing glycans for attachment and entry^37–39^. However, our findings here suggest that even sialic acid-dependent viruses, such as HCoV-OC43, also depend on cell-surface heparan sulfate to aid in their attachment to target cells, demonstrating a highly conserved role for heparan sulfate in CoV attachment. Our results highlight a conserved heparan sulfate-dependent attachment mechanism for CoVs that could be exploited to develop pan-CoV antivirals. Interactions with cellular glycans, such as HS, are critical to concentrate virions on the cell surface and enhance their specific high-affinity interactions with less abundant protein receptors, such as ACE2 for HCoV-NL63, SARS-CoV-1 and SARS-CoV-2^40,41^. Blocking these glycan-dpeendent interactions, as we show here with EGCG, disrupts infection by diverse CoVs that otherwise use different receptors and entry pathways.

To protect against future emerging CoVs, the identification of broad-spectrum entry inhibitors is a priority. Recently, lactoferrins have been described to disrupt CoV primary attachment, mediated by heparan sulfate interactions, conferring antiviral activity against multiple CoVs in vitro, demonstrating the potential for a pan-coronavirus inhibitor^42^. Similarly, bricalidin (a peptide mimetic) has been proposed to inhibit CoV infectivity by disrupting interactions with HS proteoglycans^43^. Small molecules continue to be preferred as drugs in general due to superior pharmacokinetic properties and simpler synthesis^44^. As with other green tea catechins, EGCG does not accumulate at high levels, is unstable under physiological conditions, and is rapidly metabolized^45–47^. However, our observation that this small molecule exerts pan-coronavirus activity by disrupting a conserved CoV attachment mechanism provides the basis for the development of entry inhibitors that could provide an effective prophylactic strategy to protect against future emerging CoV infections.

## Materials and methods

### Compounds

Epigallocatechin-3-gallate (EGCG, 95% pure) was purchased from Thermo Fisher Scientific (AC449010100). Heparin and N-acetylneuraminic acid were obtained from Carbosynth Ltd (YH09354 and MA0076, respectively).

### Plasmids

Lentiviral pseudoparticles were produced using plasmids encoding HIV-1 gag/pol (BEI Resources NR-52517), tat (BEI Resources NR-52518), rev (BEI Resources NR-52519), a luciferase-encoding lentiviral genome (BEI Resources NR-52516) and spike plasmids. The SARS-CoV-2 spike expression plasmid was obtained from Dr. Raffaele De Francesco (Addgene plasmid #155297). The MERS-CoV spike expression plasmid was kindly provided by Dr. Stefan Pöhlmann (Göttingen, Germany). The SARS-CoV-1 (Tor2 strain, GenBank accession no. NC_004718.3) and WIV1-CoV spike (GenBank accession no. KC881007.1) expression plasmids were synthesized by Genscript and codon-optimized for human expression. The lentiviral vector expressing human ACE2 (Dr. Sonja Best, Addgene plasmid #154981) was used with a lentiviral packaging plasmid (Dr. Didier Trono, Addgene plasmid #12260) and a mammalian expression construct for VSV-G (Dr. Bob Weinberg, Addgene plasmid # 8454).

### Cells and viruses

HEK293T/17 (ATCC ACS-4500), L929 (ATCC CCL-1), Huh7 (JCRB0403), HCT-8 (ATCC CCL-244), 17Cl-1 (BEI Resources NR-53719) and Vero E6 (ATCC CRL-1586) cells were cultured in Dulbecco's minimal essential medium (DMEM) with 10% FBS, 50 U/mL penicillin, and 50 μg/mL streptomycin at 37 °C in 5% CO_2_. A549 (BEI Resources NR-52268) cells were cultured in Hams F-12 K (Kaighn’s) medium with 10% FBS, 50 U/mL penicillin, and 50 μg/mL streptomycin, and 10 μg/mL blasticidin (for A549-ACE2 cells) at 37 °C in 5% CO_2_. Calu-3 (ATCC HTB-55) cells were cultured in minimal essential medium (MEM) with 10% FBS, 1 mM sodium pyruvate, 1X non-essential amino acid solution, 50 U/mL penicillin, and 50 μg/mL streptomycin at 37 °C in 5% CO_2_.

A549-ACE2 cells were generated by lentiviral transduction and selected for with 10 μg/mL blasticidin. The bulk population was single-cell cloned by limiting dilution and ACE2 expression of clonal populations was determined by Western blotting using a rabbit monoclonal ACE2 antibody (Invitrogen MA5-32,307) with a goat anti-rabbit IgG Alexa Fluor® 555 Conjugate (NEB 4413S) and visualized on a LICOR Odyssey CLx. Subsequently, A549-ACE2 EXT1 KO cells were generated by CRISPR/Cas9, selected for with 2 μg/mL puromycin, and single-cell cloned by limiting dilution. KO in clonal populations was confirmed by Western blotting using a rabbit polyclonal EXT1 antibody (Novusbio NBP3-03736).

HCoV-229E and HCoV-OC43 were obtained from BEI Resources (NR-52726 and NR-52725). For HCoV-229E, Huh7 cells were infected with a multiplicity of infection (MOI) of 0.01 plaque-forming units (pfu)/cell and incubated at 33 °C in 5% CO_2_ until full cytopathic effects (CPE) were observed (∼5 days post-infection). The supernatants were collected, and cellular debris was pelleted by centrifugation at 1,000 × g for 10 min. The resulting supernatant was then aliquoted, and the viral stocks were stored at − 80 °C. For HCoV-OC43, HCT-8 cells were infected (MOI of 0.01) and incubated at 33 °C in 5% CO_2_ until full CPE were observed (∼5 days post-infection). Similarly, supernatants were collected, cellular debris was pelleted by centrifugation at 1000 × g for 10 min and the resulting supernatant was then aliquoted and stored at − 80 °C.

Green fluorescent protein-expressing murine hepatitis virus (MHV-A59-GFP) was kindly provided by Dr. Volker Thiel (Institute of Virology and Immunology, Bern, Switzerland)^48^. VSV-SARS-CoV-2 S was a gift from Dr. Sean Whelan (Washington University School of Medicine in St. Louis, USA)^18^. MHV-A59-GFP and VSV-SARS-CoV-2 were propagated as described^18,48^.

### Infectivity assays

Huh7 cells (1.6 × 10^6^ cells/well in 6-well plates) were infected with approximately 100 PFU of HCoV-229E pre-exposed for 10 min at 37 °C to EGCG or dimethyl sulfoxide (DMSO) vehicle in DMEM. Inocula were removed 2 h later, and monolayers were overlaid with 1.2% carboxymethylcellulose in DMEM containing 2% FBS. Infected cells were incubated at 33 °C in 5% CO_2_ until 4 days post-infection, when they were fixed with 10% formalin and stained with 1% (wt/vol) crystal violet–10% (vol/vol) ethanol in H_2_O for counting plaques.

Huh7 and A549 cells (4 × 10^5^ cells/well in 24-well plates) were infected with approximately 200 focus-forming units (ffu) of HCoV-OC43, pre-exposed to EGCG or DMSO vehicle for 10 min at 37 °C in DMEM. Inocula were removed 2 h later, and monolayers were overlaid with 1.2% microcrystalline colloidal cellulose (Sigma-Aldrich 435,244) in DMEM containing 2% FBS. Infected cells were incubated at 33 °C in 5% CO_2_ for 3 days, then fixed and processed for immunofluorescence to detect HCoV-OC43 nucleoprotein. Cells were incubated with primary mouse IgG anti-coronavirus group antibody MAB9013 (Millipore Sigma; diluted 1:500) for 1 h at room temperature, followed by addition of secondary Alexa Fluor 488 anti-mouse IgG Fab 2 antibody (Cell Signaling Technology 4408S) for 1 h at room temperature. Foci were visualized and counted under a fluorescence microscope (Nikon Eclipse Ts2).

MHV-A59-GFP virions pre-exposed to EGCG or DMSO vehicle for 10 min at 37 °C in DMEM were used to infect L929 cell monolayers. Inocula were removed 1 h later, and monolayers were overlaid with 1.2% carboxymethylcellulose in DMEM containing 2% FBS. Infected cells were incubated at 33 °C in 5% CO_2_ for 24 h, then fixed prior to counting of GFP-positive cells under the fluorescence microscope.

VSV-SARS-CoV-2 S virions pre-exposed to EGCG or DMSO vehicle for 10 min at 37 °C in DMEM were used to infect Huh7, A549-ACE2 or Calu-3 cell monolayers in duplicate wells of a 96-well plate. Infected cells were incubated at 33 °C in 5% CO_2_ for 24 h, then fixed with 10% formalin. GFP signals were captured by Nikon Eclipse Ts2 (20X objective) processed using the Python Imaging Library to find the mean grey value, and plotted as percentage of inhibition.

In all experiments, half maximal inhibitory concentrations (IC_50_) were calculated by nonlinear regression analysis using GraphPad Prism (version 9.0; GraphPad Software, Inc.).

### Pseudoparticle entry assays

Lentiviral pseudoparticles were generated in HEK293T/17 cells by co-transfection using Lipofectamine 2000 (Invitrogen 11668–019)^49^. The CoV spike expression plasmids were co-transfected with a luciferase-encoding lentiviral genome, and plasmids encoding HIV-1 gag/pol, tat, and rev^49^. Cell culture supernatants containing CoV spike pseudotyped lentiviral particles were collected at 48 and 72 h post-transfection, pooled and filtered through a 0.45 μm filter, and stored at − 80 °C.

Pseudoparticles were incubated for 10 min at 37 °C with serially diluted EGCG and used to infect Huh7 or A549-ACE2 cells in triplicate in 96-well plates. Infectivity was determined after 3 days by luminescence following addition of BrightGlo reagent (Promega PR-E2620). IC_50_ were calculated by nonlinear regression analysis using GraphPad Prism (version 9.0; GraphPad Software, Inc.).

### Cell viability assay

Cells (50,000 per well in 96-well plate) were treated with EGCG or DMSO in DMEM and incubated at 37 °C in 5% CO_2_ for 2 h. To mimic the conditions used in the infectivity and pseudoparticle assays, media was removed, cells were washed with PBS, and supplemented with normal culture media of DMEM with 10% FBS, 50 U/mL penicillin, and 50 μg/mL streptomycin. Alternatively, cells were incubated in the presence of media containing EGCG or DMSO for 24 h or 72 h. After 72 h, cell viability was assessed by alamarBlue cell viability reagent (Invitrogen DAL1025) on a fluorescence plate reader (SpectraMax ID3 Multimode Plate reader).

### Time-of-addition experiments

Near-confluent Huh7 and A549 cell monolayers in 12 or 24-well plates were pre-treated with EGCG or DMSO vehicle for 1 h at 37 °C in DMEM. Cells were washed with DMEM and infected with 50–200 PFU of HCoV-229E or HCoV-OC43 for 2 h at 33 °C. Alternatively, Huh7 and A549 cells were first infected with HCoV-229E or HCoV-OC43 for 2 h at 33 °C. After removing the inocula, the infected cells were overlaid with 1.2% microcrystalline colloidal cellulose in DMEM-2% FBS containing EGCG or DMSO. Infectivity was assessed by plaquing efficiency or immunostaining.

### Binding assays

OC43 (4 × 10^4^ PFU; MOI 0.05) or VSV-SARS-CoV-2 (2 × 10^5^ PFU; MOI 0.5, chosen to match the conditions of the infectivity assay) were exposed for 10 min at 37 °C to EGCG or DMSO in DMEM and adsorbed onto pre-chilled Huh7 and A549 cells for 1 h at 4 °C. After three washes with cold phosphate-buffered saline (PBS), cells were lysed and RNA was isolated using the Monarch Total RNA Miniprep Kit (NEB T2010S), according to the manufacturer’s instructions. The RNA was transcribed to cDNA using the High Capacity cDNA Reverse Transcription Kit (Applied Biosystems LS4368814). Quantitative real-time PCR was performed on the QuantStudio 3 (Applied Biosystems) instrument, using PowerTrack SYBR Green Master Mix (Applied Biosystems LSA46110), with primers to detect actin (F: 5'-CTGGGAGTGGGTGGAGGC-3', R: 5'-TCAACTGGTCTCAAGTCAGTG-3') and OC43 N gene (F: 5'-CCCAAGCAAACTGCTACCTCTCAG-3', R: 5'-GTAGACTCCGTCAATATCGGTGCC-3') or VSV N gene (F: 5'-GATAGTACCGGAGGATTGACGACTA-3', R: 5'-TCAAACCATCCGAGCCATTC-3'). Following normalization to actin, the percentage of binding was expressed relative to binding of virions treated with the DMSO control.

### Heparin column chromatography

VSV-SARS-CoV-2 (10^5^ PFU) was loaded onto a 1-mL HiTrap heparin column (Cytiva 17040601) in 10 mM sodium phosphate (pH 7.4). The column was washed with 5 mL of the same buffer and eluted with heparin, EGCG, N-acetylneuraminic acid in the same buffer. Alternatively, pre-treated VSV-SARS-CoV-2 was loaded and washed with the same buffer. Still-bound virions were then eluted with 2 M NaCl in 10 mM sodium phosphate (pH 7.4). Fractions were concentrated using Amicon 100 K ultrafiltration tubes and analyzed for viral RNA.

### Statistical analysis

Data are represented as mean ± standard deviation or standard error of the mean. Data were analyzed by Prism9 (GraphPad Software) and statistical significance was determined by unpaired t-test with Welch's correction. *p* < 0.05 was considered statistically significant.

## Supplementary Information

Supplementary Information.

## Data Availability

All data generated or analysed during this study are included in this published article and its Supplementary Information files.
