# Supplementary material for: The green tea catechin EGCG provides proof-of-concept for a pan-coronavirus attachment inhibitor
Source: Sci Rep. 2022 Jul 28;12:12899. doi: 10.1038/s41598-022-17088-0 (PMC9330937; doi:10.1038/s41598-022-17088-0)

Supplementary materials for

The green tea catechin EGCG provides proof-of-concept for a pan-coronavirus attachment inhibitor

Emmanuelle V. LeBlanc and Che C. Colpitts

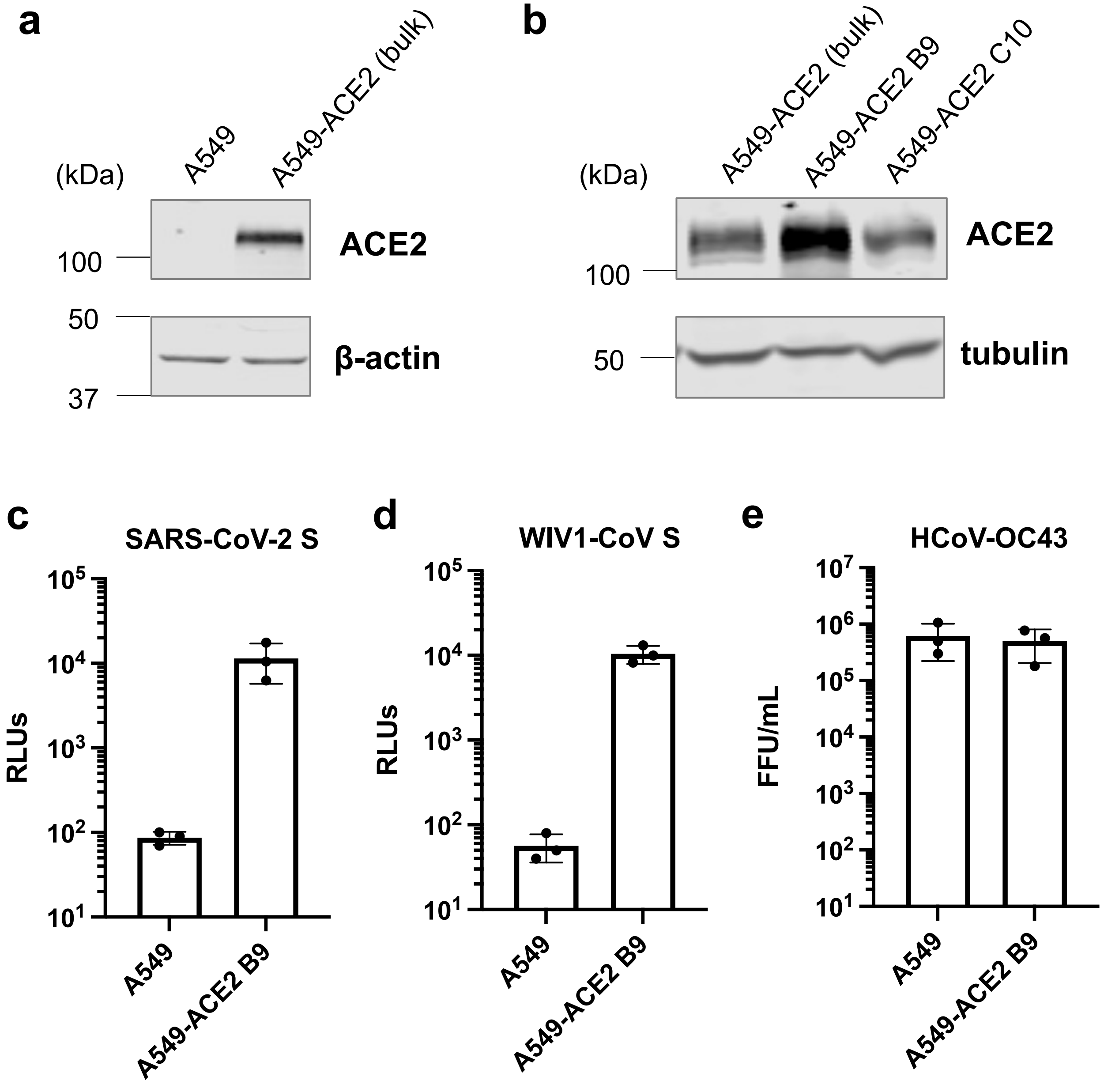

Figure S1. A549-ACE2 B9 expresses high levels of ACE2 and is susceptible to SARS-CoV-2 pseudoparticle infection. Western blot shows overexpression of ACE2 in the bulk transduced A549 cells (**a**) and in single cell clones (**b**) with A549-ACE2 B9 showing highest levels of ACE2 expression. Representative blots are shown. SARS-CoV-2 (**c**) and WIV1-CoV (**d**) pseudoparticle infection is dependent on ACE2, while HCoV-OC43 (**e**) infection is not. Mean value with standard deviation are plotted.

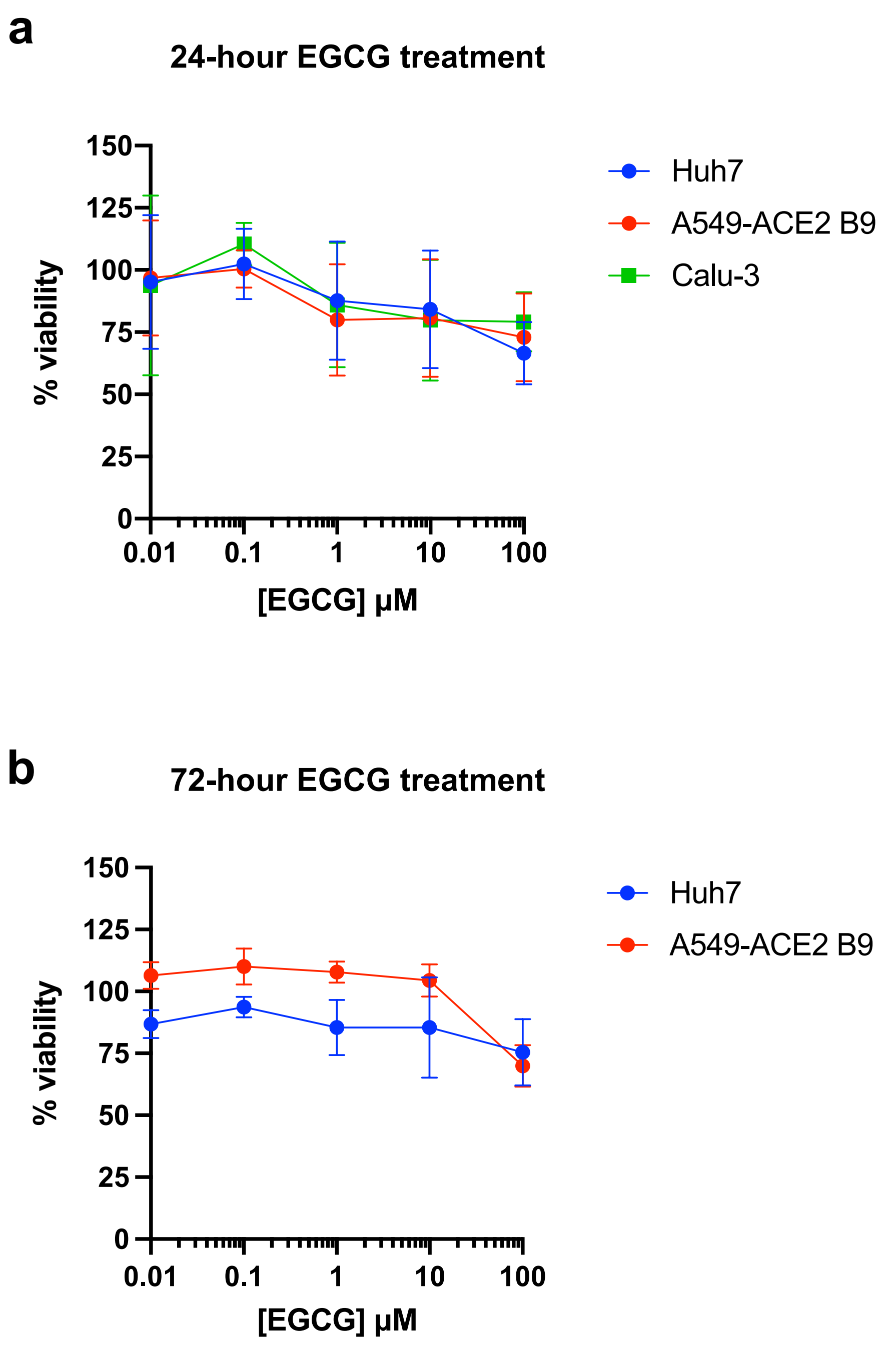

Figure S2. EGCG exposure has minor effects on cell viability. Viability of Huh7, A549-ACE2 or Calu-2 cells was assessed after 24 h **(a)** or 72 h **(b)** of EGCG exposure. Mean values with standard deviation of two independent experiments with duplicates are plotted.

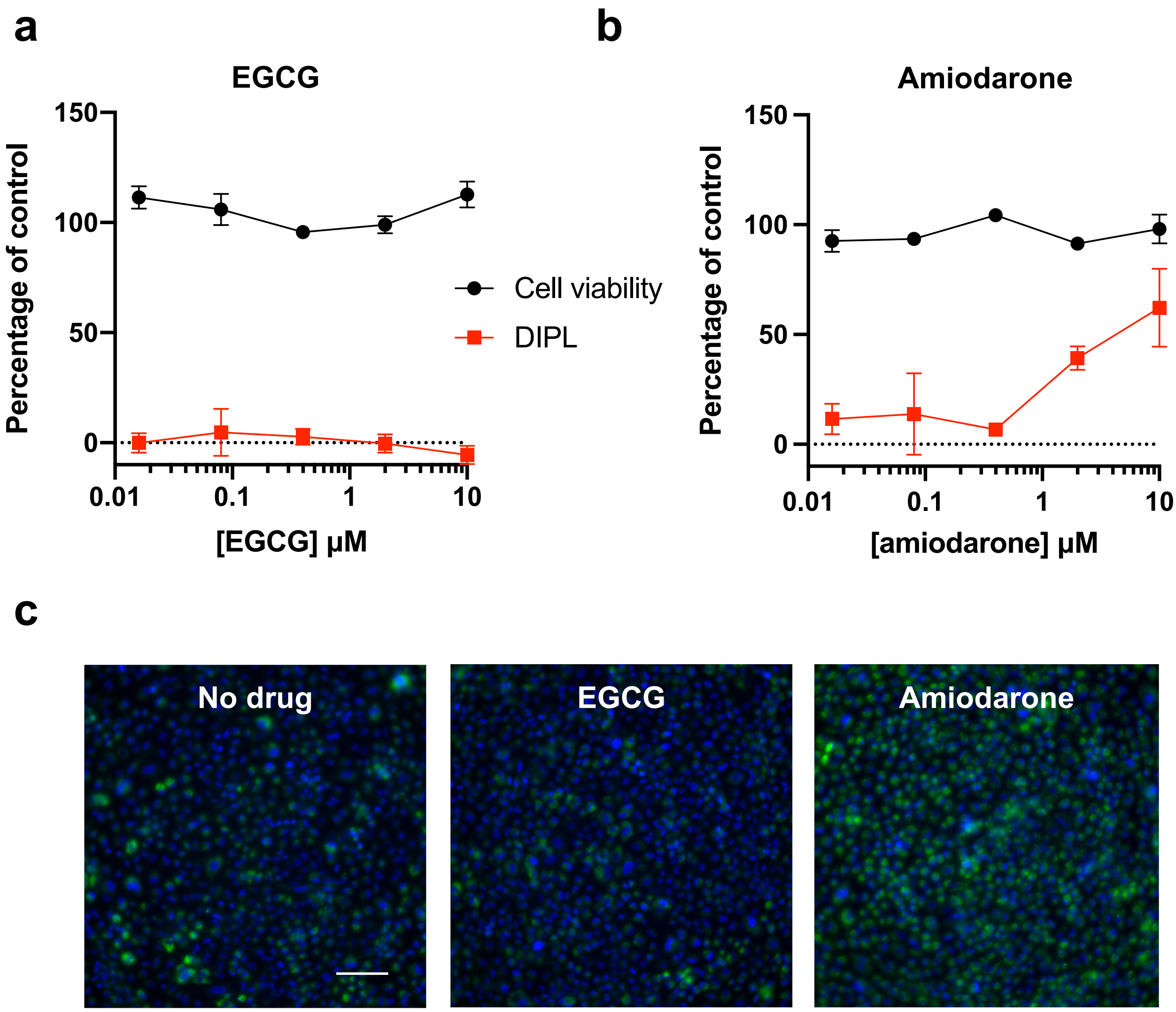

Figure S3. EGCG treatment does not induce phospholipidosis. Drug-induced phospholipidosis (DIPL) was assessed by total intensity of nitrobenzoxadiazole-conjugated phosphoethanolamine (NBD-PE) phospholipid staining of A549-ACE2 cells treated with EGCG **(a)** or amiodarone (positive control) **(b)** for 24 h. DIPL is expressed as as percentage of amiodarone-induced DIPL at 10 µM, relative to DMSO. Cell viability is expressed as percent viability of cells treated with DMSO. Mean values and standard deviation of two independent experiments (DIPL) or a single experiment (cell viability) with triplicates are plotted. **(c)** Representative images of NBD-PE (green) and Hoechst (blue) staining for no drug, or treatment with 10 µM EGCG or amiodarone, are shown. Scale bar, 100 µm.

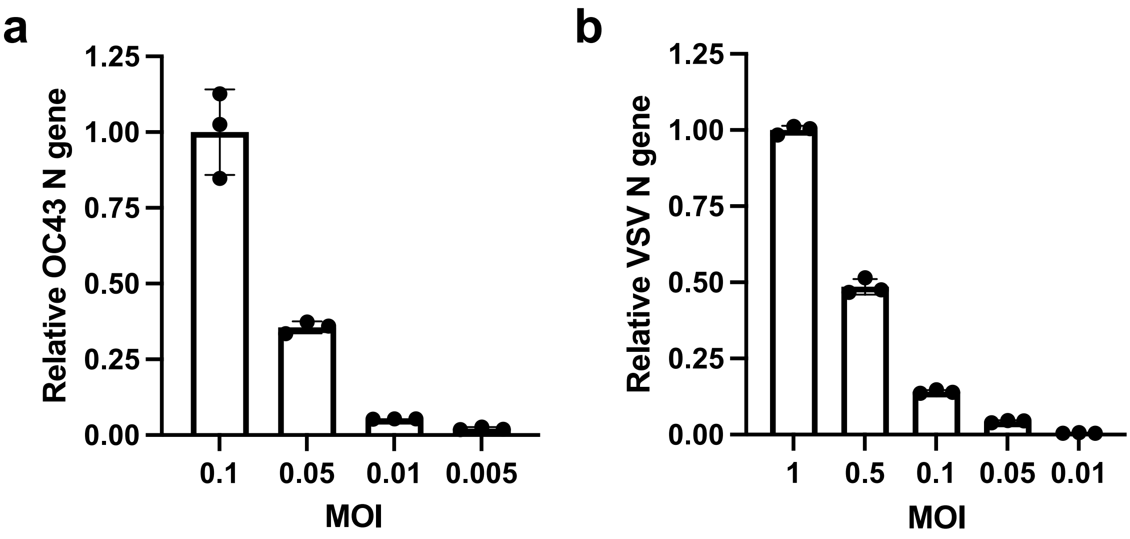

Figure S4. RT-qPCR quantification of bound virions. Pre-chilled Huh7 cells were inoculated with HCoV-OC43 (**a**) or VSV-SARS-CoV-2 (**b**) at indicated MOI for 1 hour on ice. Attached virus was quantified by RT-qPCR after washing with PBS three times. Mean values with standard deviation from qPCR triplicates are plotted.

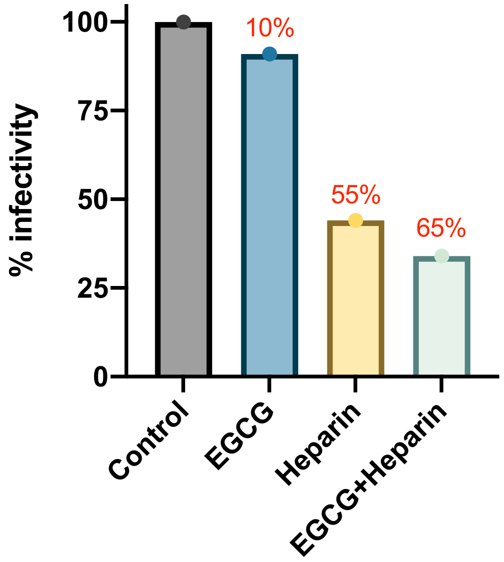

Figure S5. Treatment with EGCG and heparin in combination exhibits an additive effect against HCoV-OC43 infectivity. Pre-treatment of HCoV-OC43 virions with EGCG, heparin, or a combination of EGCG (0.5 µM) and heparin (10 µg/mL). The combination treatment reduced infection of Huh7 cells in an additive manner. Percent reduction in infectivity is shown in red font. One representative experiment is shown.

Full-length blots

From Figure 4b.

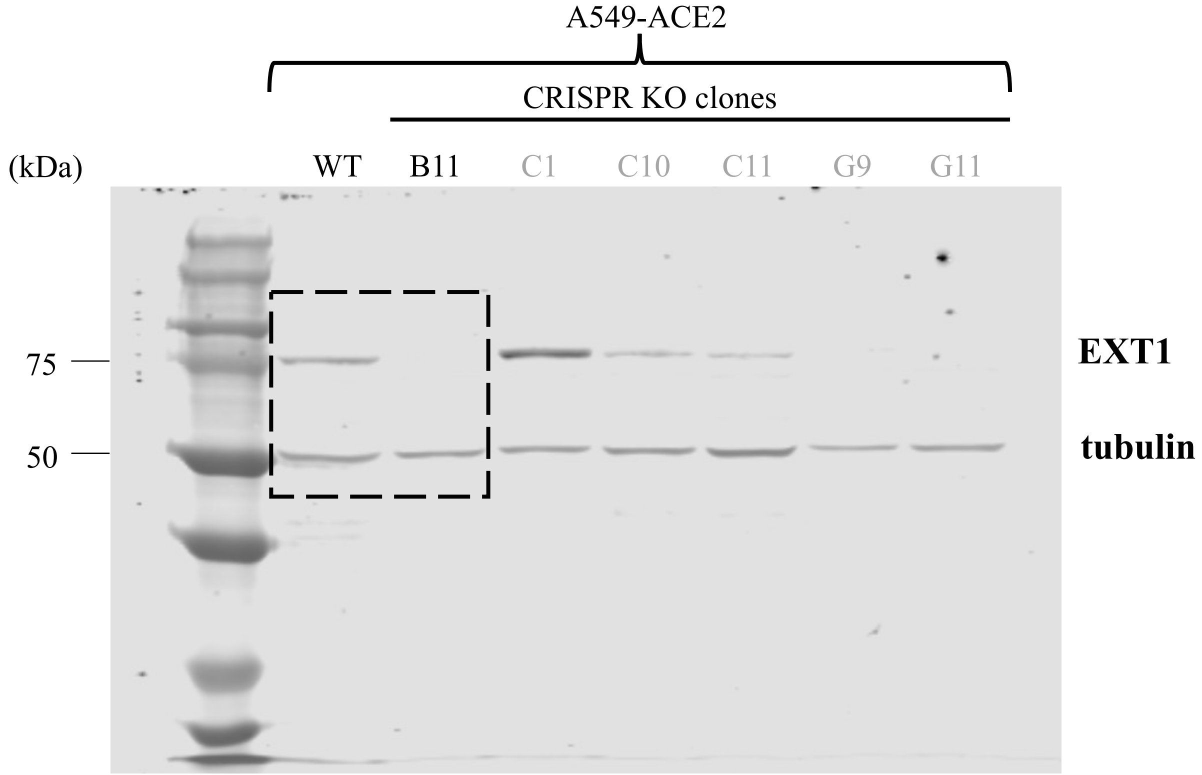

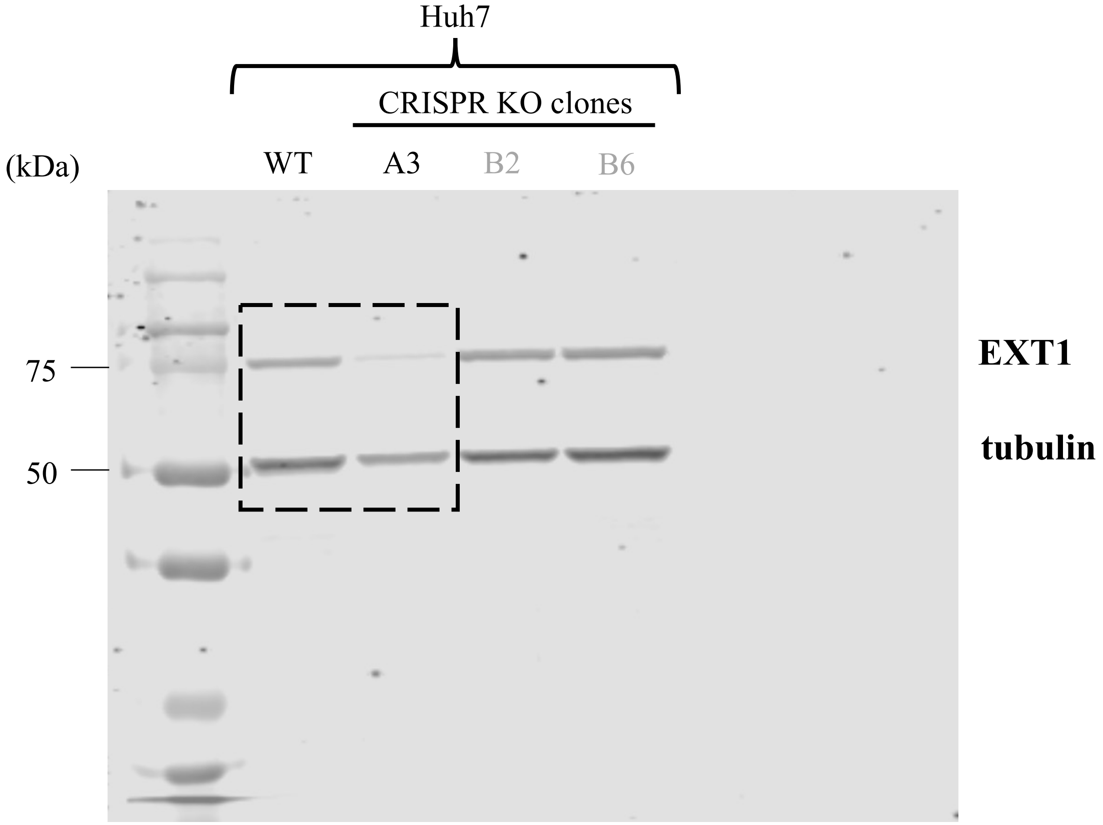

From Figure S1a.

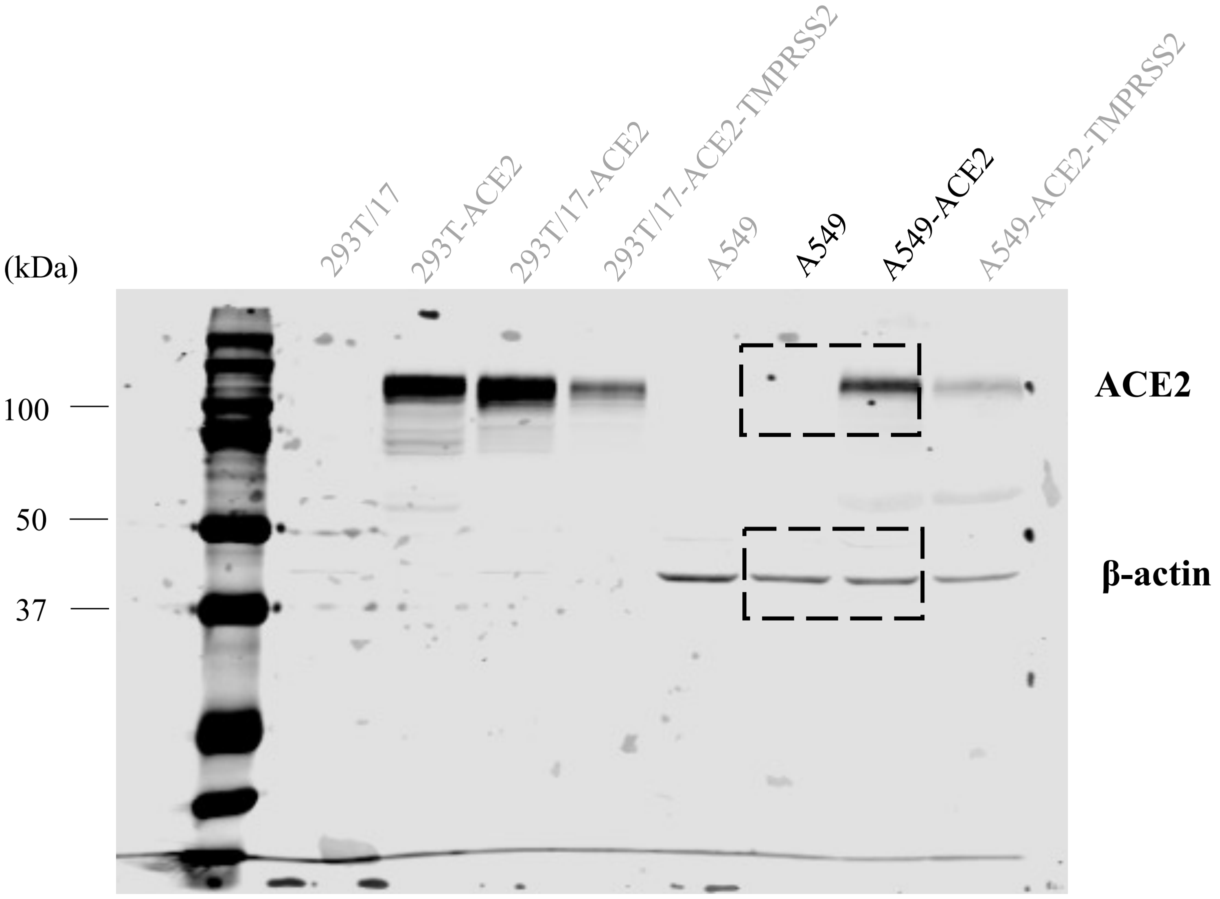

From Figure S1b.

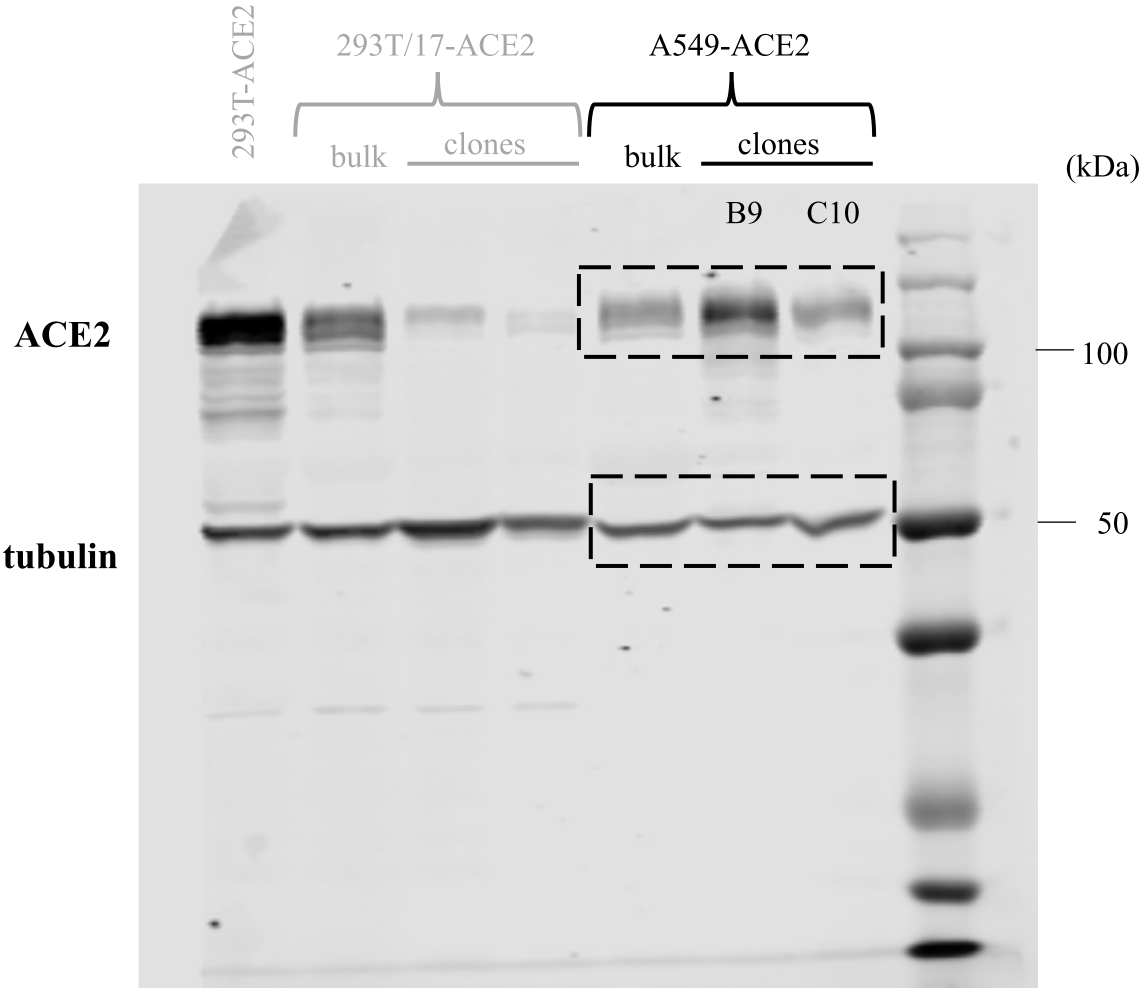

Supplement: Supplementary file 1 — Supplementary Information. [file 41598_2022_17088_MOESM1_ESM.pdf]
